# Dye-sensitized solar cells based on a push-pull zinc phthalocyanine bearing diphenylamine donor groups: computational predictions face experimental reality

**DOI:** 10.1038/s41598-017-15745-3

**Published:** 2017-11-15

**Authors:** Riccardo Milan, Gurpreet Singh Selopal, Marco Cavazzini, Simonetta Orlandi, Rita Boaretto, Stefano Caramori, Isabella Concina, Gianluca Pozzi

**Affiliations:** 1 Department of Information Engineering, University of Brescia, Via Valotti, 9 – 25131 Brescia, Italy; 2 CNR-INO SENSOR Laboratory, via Branze, 45 – 25123 Brescia, Italy; 3 Schulich Faculty of Chemistry, Technion−Israel Institute of Technology, 32000 Haifa, Israel; 4 Institute of Fundamental and Frontier Sciences, University of Electronic Science and Technology of China, Chengdu, 610054 P. R. China; 5 Institut National de la Recherche Scientifique, Centre Énergie, Matériaux et Télécommunications, 1650 Boul. Lionel Boulet, Varennes, QC J3X 1S2 Canada; 6 Institute of Molecular Science and Technology, ISTM-CNR, Via Golgi 19, 20133 Milano, Italy; 7 Department of Chemical and Pharmaceutical Sciences, University of Ferrara, Via Borsari 46, 44121 Ferrara, Italy; 8 Division of Materials Science, Department of Engineering Sciences and Mathematics, Luleå University of Technology, 971 87 Luleå, Sweden

## Abstract

Computational studies have suggested that the integration of secondary amine as donor groups in the structure of unsymmetrical zinc phthalocyanine (ZnPc) should have positive effects on photovoltaic performance, once the molecule is integrated as light harvester in dye sensitized solar cells (DSSCs). Aiming at obtaining experimental confirmation, we synthesized a peripherally substituted push-pull ZnPc bearing three electron donating diphenylamine substituents and a carboxylic acid anchoring group and integrated it as sensitizer in TiO_2_-based DSSCs. Detailed functional characterization of solar energy converting devices resulted in ruling out the original hypothesis. The causes of this discrepancy have been highlighted, leading to a better understanding of the conditions for an effective design of push-pull diarylamino substituted ZnPcs for DSSCs.

## Introduction

**Figure 1:**
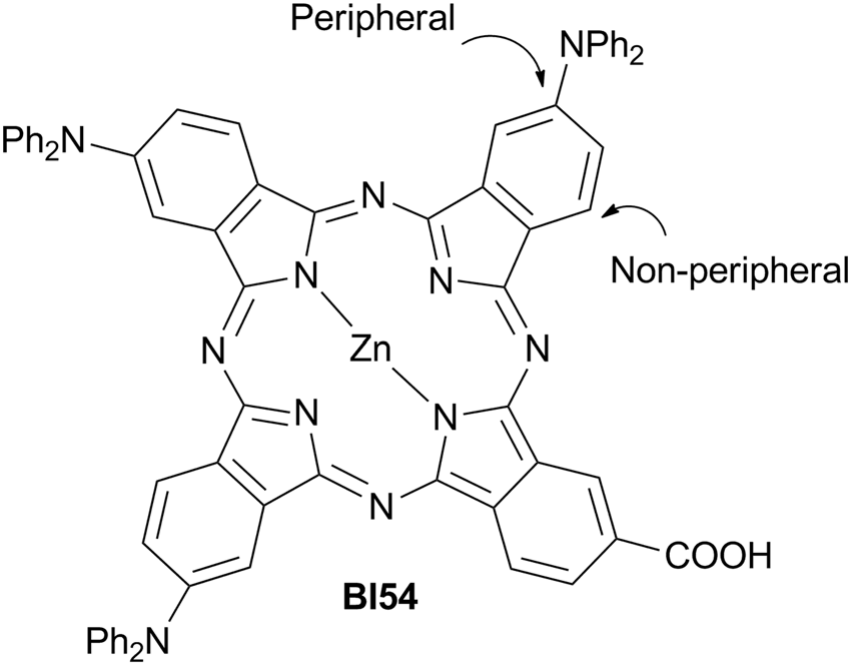
Peripherally substituted A3B ZnPc **BI54** bearing diphenylamino donors. Only one positional isomer is shown.

Many research endeavors to spur the efficiency in solar energy conversion of dye sensitized solar cells (DSSCs)^1^ are currently directed to the development of optimized light harvesters, capable of exploiting the whole range of the solar spectrum, including the infrared portion lost in present silicon-based PV technology, in order to eventually obtain the so-called panchromatic sensitization. Investigations from diverse standpoints, spanning from the comprehension of the underlying fundamental phenomena to the assessment of the life cycle and economic effectiveness of these devices, are as well pursued, but special attention is paid to the design of new photosensitizers that could ensure optimal photovoltaic parameters and acceptable lifetime service to DSSCs^2^. Ruthenium-based sensitizers had a prominent role in early developments of DSSCs and are still widely employed, but they show only moderate light harvesting capability, in particular in the red/near IR spectral regions of the solar radiation^3^. In this respect, other families of dyes, such as phthalocyanine derivatives^4^, offer better perspectives especially in view of the architectural integration of DSSCs in energy efficient buildings. Indeed phthalocyanines (Pcs) are thermally, photochemically and electrochemically stable compounds, with unparalleled light-harvesting capability in the far-red and near-IR spectral regions where a substantial solar fluence occurs. These peculiar properties can be fine-tuned by relatively simple synthetic modifications^5^. The electronic absorption spectrum of Pcs shows two main bands at 300–400 nm (B band) and 620–700 nm (Q band with extinction coefficients in the order of 10^5^ M^−1^ cm^−1^). These bands can be shifted or broadened depending on the presence and nature of substituents in the peripheral and non-peripheral positions (see Fig. 1) of the macrocycle. Other relevant characteristics, as the HOMO-LUMO energy level and the distribution of the electronic density, can be varied following the same strategy. Finally, metallation of the macrocycle also allows to tune the behavior of Pc-based photosensitizers and Zn(II) complexes of Pcs (ZnPcs) with their long-lived singlet excited states proved to be remarkably useful in DSCCs^6^.

The most successful ZnPcs sensitizers reported so far are unsymmetrically substituted molecules characterized by the presence of identical bulky, electron-donating groups on three of the four isoindole subunits (referred to as A) which constitute the Pc macrocycle, while the fourth one (referred to as B) bears one or more anchoring groups able to interact with the metal oxide semiconductor^7,8^. This A3B molecular design aims to combine steric suppression of dye aggregation, favorable electronic push-pull effects and optimized binding mode of the dye to the surface of the semiconductor layer. It was originally suggested by Nazeeruddin, Torres and coworkers, who were able to achieve power conversion efficiencies up to 4.1% in the case of peripherally substituted A3B ZnPcs bearing *tert*-butyl donors and -COOH anchoring groups^9–11^. The advent of A3B ZnPcs featuring bulky, electron rich aryl substituents rotated with respect to the macrocycle plane represented a second significant step forward towards efficient DSSCs based on Pcs^12,13^. For instance, Mori, Kimura and coworkers have shown that power conversion efficiencies up to 6.4% under simulated one-sun illumination can be obtained using a peripherally substituted A3B ZnPc bearing electron-rich 2,6-bis(*n*-butoxy)phenoxy groups on A and one electron withdrawing –COOH group on B^14^. Despite the steady progress made in the field, the full potential of ZnPcs as photosensitizers in DSSCs has yet to be realized and further improvements are conceivable^7,8^. In particular, the application of predictive computational techniques for the rational molecular design of efficient unsymmetrical push-pull ZnPcs has been proposed^15–17^. According to these theoretical studies, the use of -NH_2_, -NHR, -NR_2_, NHAr or -NAr_2_ donor groups instead of alkyl or phenoxy donor groups should improve the photovoltaic performance of unsymmetrical A3B ZnPcs thanks to higher LUMO levels, smaller energy gaps and red-shifted absorption bands. New absorption bands are also expected to emerge in the 400–600 nm region for A3B ZnPcs bearing diphenylamino substituents, which should make them good candidates as panchromatic sensitizers. In the attempt to provide experimental verification to these so far untested predictions, we have synthesized the A3B peripherally tetrasubstituted ZnPc **BI54** (Fig. 1) fitting the theoretical model. This molecule has been integrated in DSSCs based on mesoscopic TiO_2_ as electron transport material and a careful functional characterization of the devices has been carried out.

## Results and discussion

We recently developed an efficient procedure for the preparation of 4-(diarylamino)phthalonitriles and demonstrated their use as precursors of symmetrical A4 ZnPcs^18^. Zn(II)-templated cyclization performed on a mixture of the simplest of these building-blocks, 4-(diphenylamino)phthalonitrile **1**, and pentyl 3,4-dicyanobenzoate **2** (molar ratio 3:1) followed by hydrolysis of the ester functionality afforded the A3B ZnPc **BI54** in 12% overall yield (Scheme S1, Supporting Information). Having proved the synthetic feasibility of A3B ZnPcs with diarylamino substituents, we next examined the possible use of **BI54** as a sensitizer in DSSCs.

**Figure 2:**
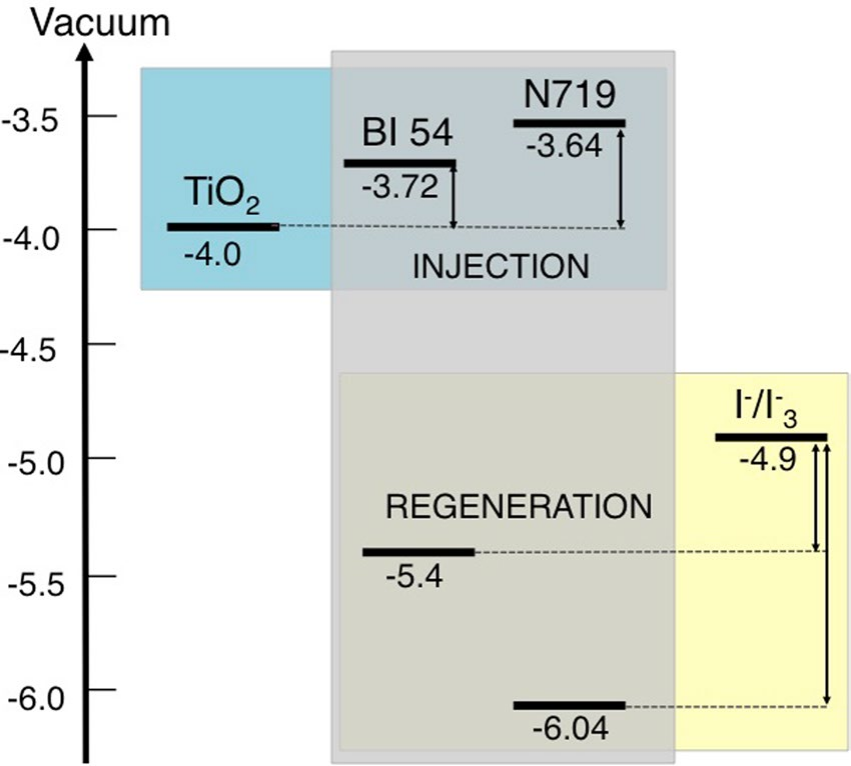
Energy alignment for the components of the solar energy converting devices. **N719** is used as reference.

The architecture of a DSSC requires the favourable energy alignment of all functional components the electrochemical circuit, namely the electron transport material (ETM), the sensitizer and the redox couple responsible for the regeneration of the photooxidized dye^1^. The energy levels of the HOMO-LUMO orbitals of the new dye were thus assessed by electrochemical measurements combined with the analysis of the optical properties (See SI, Figures S1, S2 and S3 and Table S1). The heterogeneous electron transfer kinetics at glassy carbon interfaces resulted in quasi-reversible, diffusion-limited waves, with chemical reversibility attained for scan speed > 100 mV/s. Figure 2 sketches the energy alignment of the semiconducting metal oxide (MOx) used as ETM in this work (TiO_2_) with **BI54**. The energy levels of an efficient Ru-based dye (**N719**), widely employed in DSSCs, are also reported for comparison. **BI54** presents the needed requirements to be applied as light harvester in DSSCs, with energy levels of about −5.4 eV for the HOMO orbital and −3.72 eV for the LUMO orbital, which would imply a thermodynamically favoured electronic injection from the dye to the TiO_2_ conduction band (CB) (ΔG_INJ_^BI54-TiO2^ ~0.28 eV).

**Figure 3:**
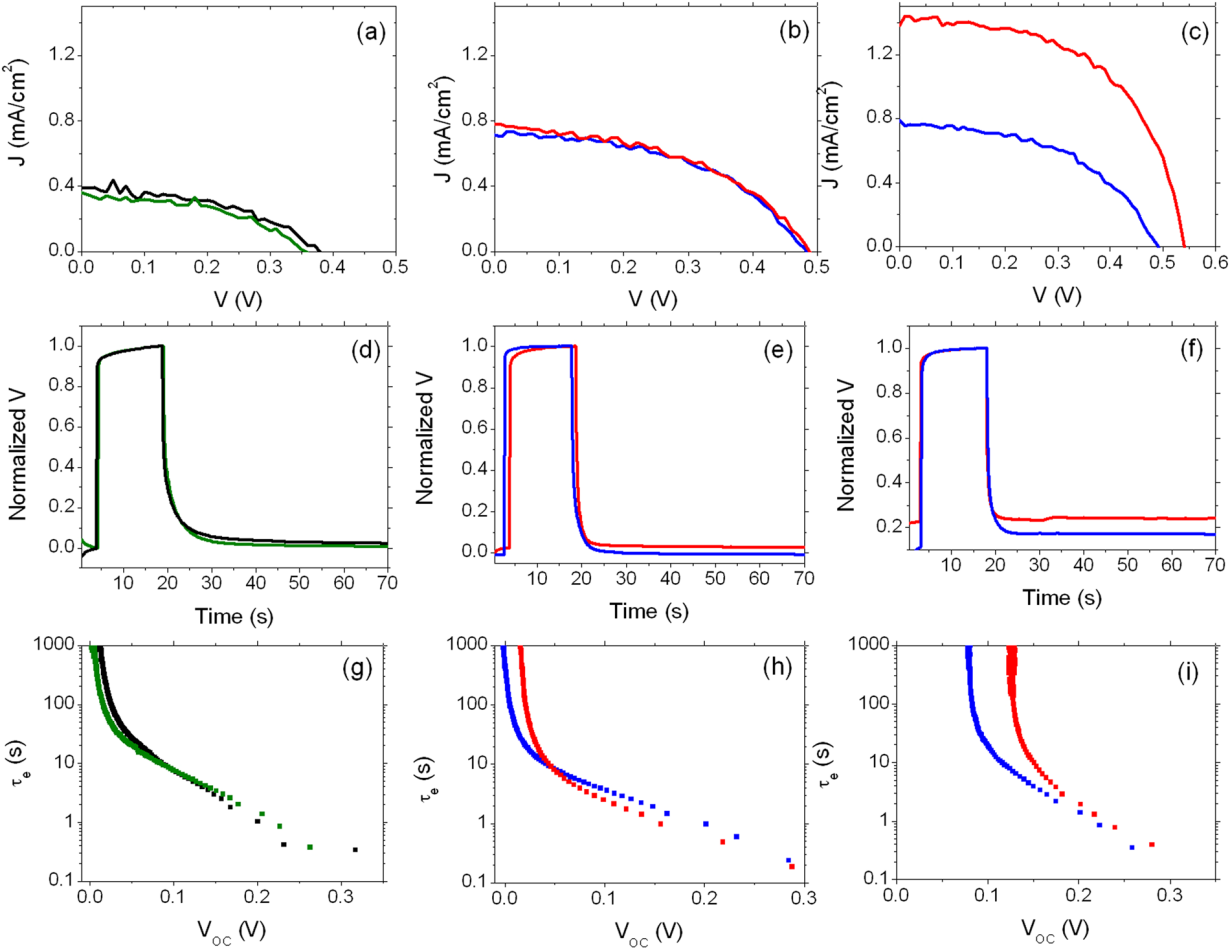
Analysis of DSSCs using **BI54** dye as light harvester. (**a**), (**b**) and (**c**): *J-V* curves of DSSCs sensitized for 20 h, 13 h and 5 h, respectively. (**d**), (**e**) and (**f**): normalized open circuit photovoltage decay (sensitization time: 20 h, 13 h, 5 h). (**g**), (**h**) and (**i**) calculated electron lifetime (sensitization time: 20 h, 13 h, 5 h, respectively). Black and green lines in (**a**), (**d**) and (**g**) refer to photoanodes made by TiO_2_ nanoparticles and MWCNT-TiO_2_, respectively. Blue and red lines in (**b**), (**c**), (**e**), (**f**), (**h**), (**i**) refer to device sensitized with **BI54** dye alone and **BI54** dye with DCA, respectively.

Two kinds of ETM have been prepared and tested as photoanodes: bare commercial TiO_2_ nanoparticles and composite multi-walled carbon nanotubes-TiO_2_ nanoparticles (MWCNT-TiO_2_), which have been reported to enhance the overall device functional performances^19^. Fig. 3a, d and g show the functional characterization of two typical DSSCs whose photoanodes are made by TiO_2_ and MWCNT-TiO_2_ sensitized for 20 hours, which is the commonly used soaking time for Ru-based dyes.

Low photocurrent density values (in both cases lower than 0.4 mA/cm^−2^) were recorded and a depleted open-circuit voltage (V_*OC*_) was as well observed (350 mV and 370 mV for the devices using bare TiO_2_ and MWCNT-TiO_2_ as ETM, respectively). Moreover, no significant difference between the photoanodes with and without MWCNTs was recorded. Analysis of open circuit voltage decay (OCVD) highlights slow charging kinetics: after 20 s irradiation the V_*OC*_ value was not reached (Fig. 3d), indicating slow charge accumulation, accompanied by electron recombination (both with **BI54** ^+^ and I_3_^−^). Decay dynamics were essentially the same for both devices. As expected from OCVD observation, electron lifetime (Fig. 3g) is the same in all the voltage range analyzed for both devices.

Pcs aggregation due to π-π stacking has been previously reported as a serious issue affecting these molecular dyes^20–22^, with consequent lowering of associated device performances: co-adsorption of a molecular spacer on TiO_2_ has then been proposed as an effective countermeasure. Deoxycholic acid (DCA) was thus added during the sensitization of bare TiO_2_ photoanodes and the soaking time reduced from 20 to 13 h. Decreased sensitization time resulted in dramatically enhanced short-circuit current density (J_*SC*_) values (Figure 3b and Table 1, Entry 3 vs 1), which were almost doubled, compared with those recorded for devices obtained after 20 hours soaking. However, the presence of DCA was found to be not significant in terms of enhanced solar energy conversion efficiency (PCE), which was calculated as high as 0.17% and 0.18% with and without DCA. A further shortening of the photoanode staining, down to 5 hours resulted in no improvement of cell efficiency *in the absence* of DCA (Fig. 3c, blue line). Actually, when no DCA is co-adsorbed on TiO_2_ photoanode the V_*OC*_ value is not changed with respect to 13 hours soaking and only a slight increase in J_*SC*_ value, from 0.715 mA/cm^−2^ to 0.764 mA/cm^−2^ can be appreciated (Table 1 Entry 5 vs 3).

**Table 1:** Functional parameters of solar energy converting devices based on TiO_2_ uploaded with dye BI54 for different sensitization times.

Entry	Sensitization time (h)	J_*SC*_ (mA/cm^2^)	V_*OC*_ (mV)	FF (%)	PCE (%)
1^a^	20	0.361	350	47	0.060
2^b^	20	0.392	370	47	0.068
3^a^	13	0.715	480	49	0.17
4^a,c^	13	0.777	480	47	0.18
5^a^	5	0.764	480	51	0.18
6^a,c^	5	1.376	540	58	0.43
7^a^	4	3.02	520	58	0.92
8^a,c^	4	3.39	510	66	1.13

^a^Bare TiO_2_; ^b^ MWCNT-TiO_2_; ^c^ DCA added.

These findings indicated once more the critical role played by phthalocyanine aggregation on photoanode surface, even for reduced uptake times. Indeed, by decreasing the soaking time to 5 hours *and* co-adsorbing DCA (Fig. 3c, red line), a remarkable enhancement of device working parameters (J_*SC*_ = 1.376 mA/cm^2^, V_*OC*_ = 540 mV, PCE = 0.43%) is recorded, corresponding to an increase of 80%, 13% and 140%, respectively, with respect to the analogous device with no DCA (Table 1, Entry 6 vs 5).

The increase of V_*OC*_ is ostensibly related to the negative shift of Fermi level, which may be induced upon the insertion of co-absorbent in the dye^23^, while the enhancement in the photocurrent density J_*SC*_ is ascribable to the increased electron injection and charge collection efficiency^24^.

The improvements achieved by the combined reduction of sensitization time and use of DCA as co-adsorbent also involve the electron lifetime as depicted in Fig. 3g–i. For devices fabricated in the absence of DCA, no difference is observed using sensitization times of 20 and 13 hours (Fig. 3g, black line, and Fig. 3h, blue line, respectively). For 13 hours sensitization (Fig. 3h) the presence of DCA results in a small, but appreciable difference. Finally, the electron lifetime is highly enhanced by the presence of DCA for 5 hours sensitization (Fig. 3i).

**Figure 4:**
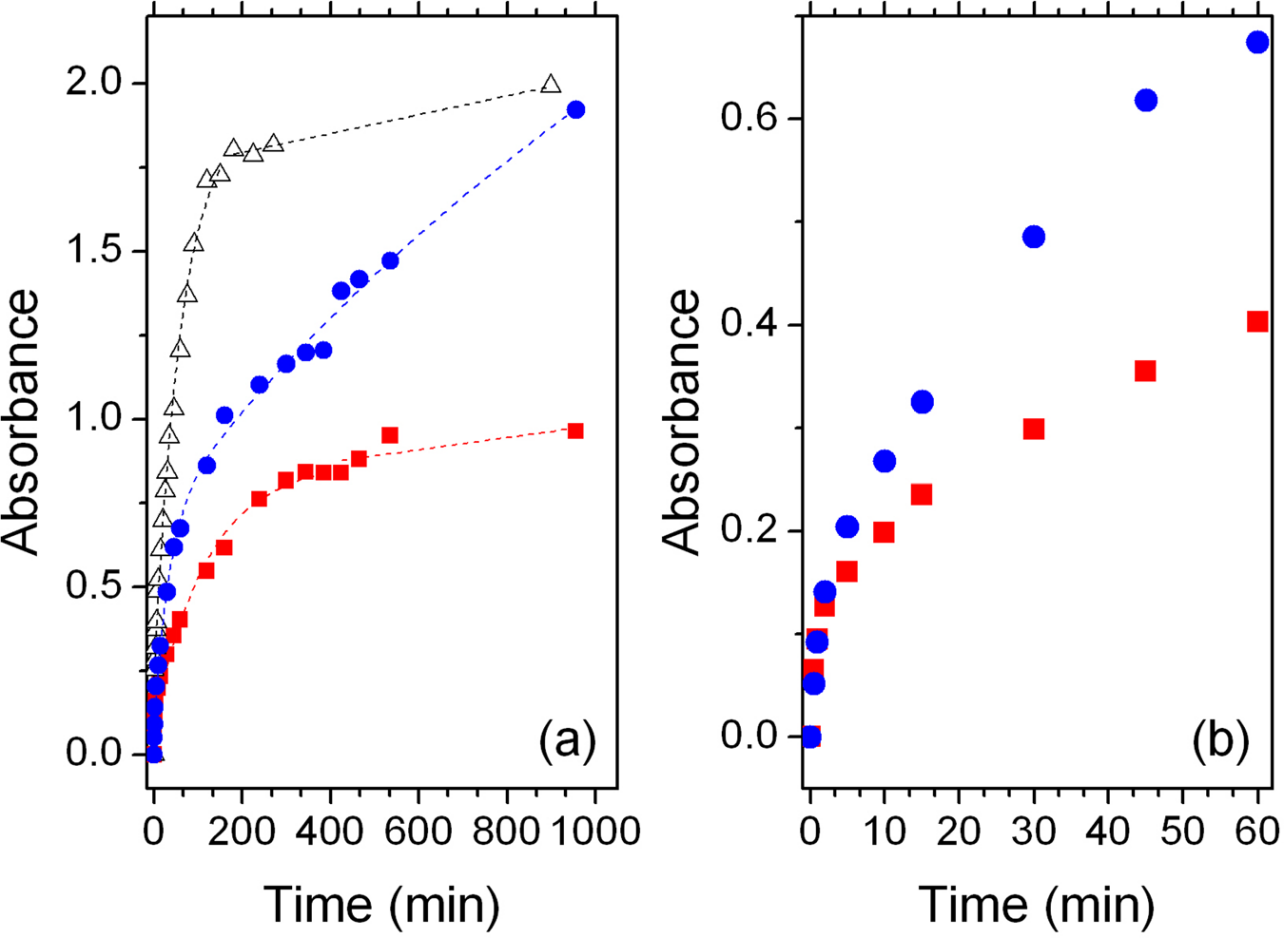
Dye uptake analysis: (**a**) Overview of dyeing process and (**b**) detail for uptake of **BI54** in the first hour. Blue circles = **BI54** without DCA; red squares = **BI54** with DCA; empty triangles = **N719**. Markers are experimental data, lines are fitting curves as discussed in the text.

In order to gain a deeper insight on the relationship between device performances and sensitization time with/without DCA, a kinetic study of the uptake of **BI54** from DCM solutions has been carried out by following the variation of optical density of the photoanode at 725 nm (Fig. 4a). The commercially available Ru-based dye **N719**, for which a detailed uptake kinetic analysis (monitored at 500 nm) has been previously reported^25^, was used as a reference.

Dye uptake on MOx surface is usually a two-step process that can be properly analyzed as a fast exponential growth followed by a second slower step, possibly featuring a pseudo 0-order kinetic, moving towards a plateau.

The first part of the uptake curve can be suitably fitted by the relationship:

1 $$[TD]={[T]}_{0}(1-{e}^{-{k}_{1}^{I}t})$$

Where [T]_0_ refers to the sites originally available on TiO_2_ for dye uptake and TD to the sites occupied (dye onto TiO_2_). The second part of the uptake curve is properly fitted by linear regression, and follows the relationship:

2 $$[TD]={[TD]}^{I}+{k}_{2}t$$

where k_1_ and k_2_ are the kinetic constants and [TD]^I^ refers to the dye concentration at the time in which the curve changes from fast to low uptake.

**BI54** does not reach a plateau in the uptake curve, not even after 25 hours impregnation. The analysis of apparent kinetic constants (Table 2) reveals other significant details. In the absence of DCA, the initial rate of **BI54** anchoring on TiO_2_ sites (exponential part of the uptake curve) is comparable to that shown by **N719**, but the second, linear step is dramatically faster (almost four times). A different trend is observed in the case of addition of DCA. The uptake curve of **BI54** reaches a plateau after a few hours of soaking, thus confirming the role exerted by DCA, which competes with the dye for TiO_2_ anchoring sites. This can be as well inferred by the much slower increase of the exponential growth, well visible in Fig. 4a, and by the comparison of the apparent kinetic constants (Table 2). The presence of DCA, indeed, slows down the uptake kinetics by occupying available sites on TiO_2_ nanoparticles surface, thus limiting the amount of **BI54** molecules anchoring on the photoanode: the rate of anchoring in the first step is 1.5 times slower in presence of DCA, while the rate in the second step is decreased of almost six times.

**Table 2:** Kinetics data for dye uptake onto TiO_2_.

Dye	*k*_1_’ ^a^ (s^−1^) × 10^3^	*k*_2_^b^ (Ms^−1^) × 10^3^
**BI54**	13.06	1.03
**BI54** (+DCA)	8.144	0.1805
**N719** ^c^	16.15	0.2808

^a^Determined by fitting of eqn (1); ^b^Determined by fitting of eqn (2); ^c^Values retrieved from ref^25^.

The kinetics of the anchoring process of **BI54** is affected by the presence of DCA even at very reduced dyeing times (Fig. 4b): this provides a further evidence that DCA competes very efficiently with **BI54** for TiO_2_ available anchoring sites^24^, a critical factor for reducing photogenerated charge recombination in solar energy converting devices^23^.

**Figure 5:**
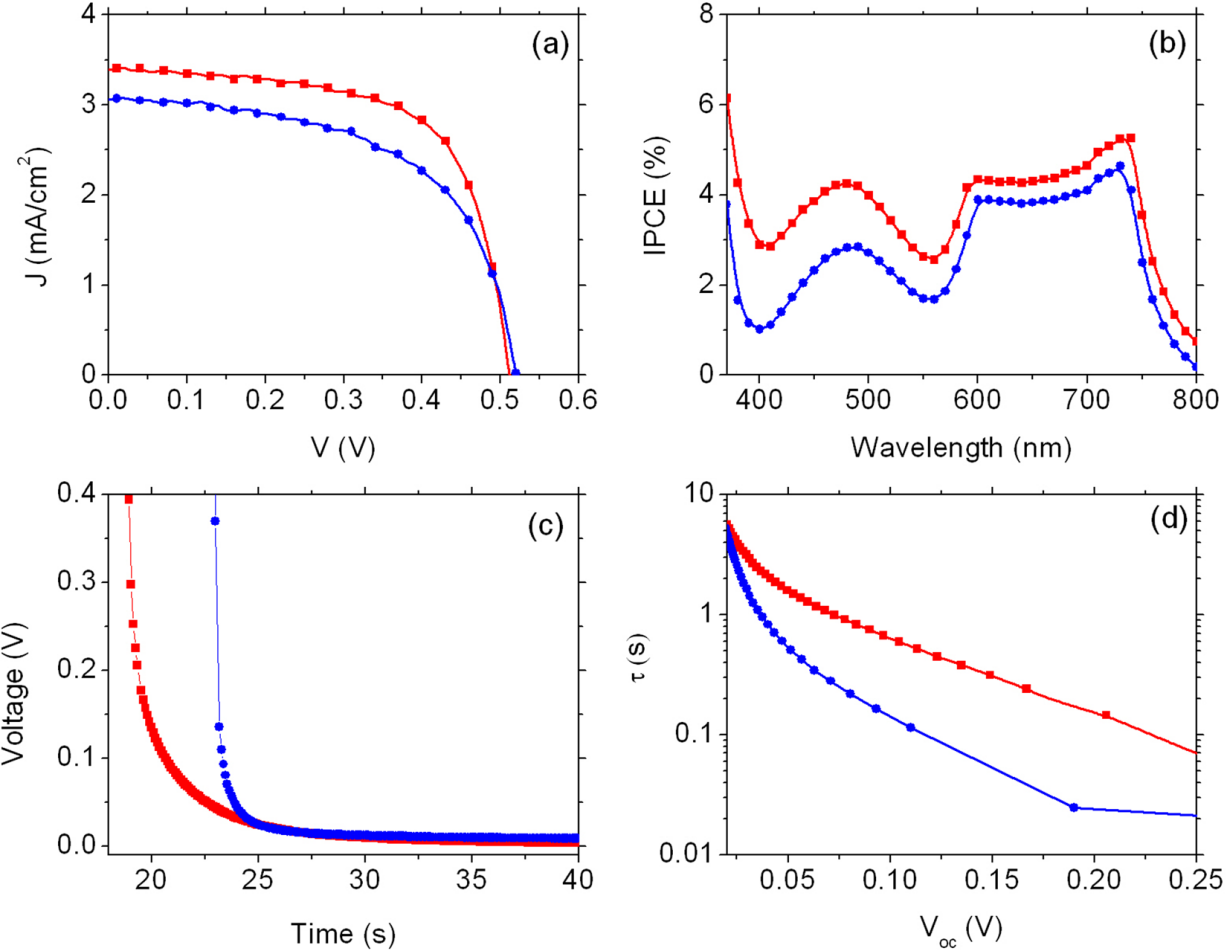
Functional characterization of DSSCs using **BI54** dye as light harvester with (red markers) and without DCA (blue markers) (sensitization time: 4 h). (**a**) *J−V* characteristics; (**b**) IPCE; (**c**) photovoltage decay and (**d**) electron lifetime. Markers are experimental points, lines are guide for the eye.

A final batch of DSSCs has been then fabricated using a sensitization time of 4 hours and analyzed. In the absence of DCA, a significant improvement of PCE (from 0.18% to 0.92%) with respect to 5 hours soaking, is observed. Also under these conditions, the presence of DCA clearly contributes to enhance cell efficiency, mostly by enhancing the photocurrent density (from 3.02 to 3.39 mA cm^−2^), while both V_*OC*_ and fill factor (FF) present similar values (Fig. 5a and Table 1, entries 7 and 8). The increased photocurrent density results in an improvement of the photoconversion efficiency of about 23% (from 0.92% to 1.13%). Consistent with *J-V* curves, incident photon to electron conversion efficiency (IPCE) spectra, reported in Fig. 5b, show an enhancement in all the visible range when DCA is present. In particular IPCE values are increased in the NIR region (730 nm) from 4.6% to 5.2% (14%) and at lower wavelengths (480 nm) from 2.8% to 4.2% (50%). IPCE data recorded in cells having a comparable optical density indicate that the factor ϕ_inj_ × η_collection,_ where ϕ_inj_ is the injection quantum yield and η_collection_ is the electron collection efficiency across the photoanode, is improved. The increased quantum yield may result from reduced aggregation, which would otherwise lead to excited state quenching or exciton annihilation. It should be herein reminded that only the first monolayer of adsorbed dye is effective in generating useful photocharges, while subsequent adsorption, via dispersive forces, of dye molecules to form molecular aggregates decoupled from the titania surface is detrimental, resulting in parasitic light absorption and quenching. OCVD analysis, reported in Fig. 5c, shows a drastically reduced drop of voltage for the cell in which DCA is used. By fitting the OCVD curves with an exponential function, the time decay constants may be estimated at 0.45 s and 2.18 s for the cells without and with DCA, respectively, indicating an increase of almost five times of the decay rate when **BI54** is left free to aggregate on TiO_2_.

The smoother V_*OC*_ decay is reflected in an increased electron lifetime (Fig. 5d): after 0.10 s the voltage is quite reduced (0.11 V) if DCA is not employed, while it is twice this value (0.23) when DCA is present.

Open circuit photovoltage observed in all the investigated DSSCs show reduced values as compared with the expectation for TiO_2_ based cells in the presence of the iodide redox couple. Such a decrease may origin from either a decrease in the band offset or an increased recombination rate constant. A shift in the band edge (which would alter the relative energy positions of TiO_2_ CB and standard redox potential of the electrolyte redox couple that determines the V_*OC*_) can be estimated through electrochemical impedance spectroscopy (EIS) by a shift in the chemical capacitance of the cell as a function of voltage.

**Figure 6:**
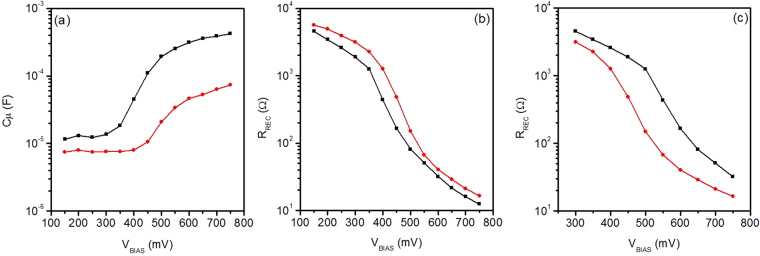
(**a**) Chemical capacitance, (**b**) as collected recombination resistance and (**c**) recombination resistance calculated for the same electron density of DSSCs using **BI54** (red line) and **N719** (black line) as sensitizer.

Figure 6a shows the chemical capacitance of DSSCs sensitized with **BI54** (with DCA) and **N719** (used as reference). In the low potential region (100–300 mV), the trend of the internal capacitances of the cells is quite constant and is originated by the interfaces of the platinum counter electrode and of the FTO back contact of the photoanode^26^. In the high potential region the chemical capacitance dominates the capacitive effects inside the cells, showing a linear trait on the logarithmic scale. The chemical capacitance is directly related to the density of electronic states (DOS) in the metal oxide^27,28^. The trend of the chemical capacitance could then give information about the displacement of the CB of the metal oxide. Since the thickness and the porosity of the oxide are the same for both cells, the effect on the CB is ascribable to the interaction between the dye and the metal oxide^27–33^.

The net effect in the case of **N719** is the downward shift of the E_C_ (by about 150 mV), resulting in an enhancement of the charge injection from the Ru(II) dye to the metal oxide. This contributes to explain the lower photogenerated current observed for **BI54**, as compared with **N719**. Moreover, the slopes of the chemical capacitance vs potential curves are different for the two sensitizers, and in particular it appears less steep for **BI54**. As shown by Barea *et al*. this means that the interaction between **N719** and the TiO_2_ modify the trap states^34^.

**Figure 7:**
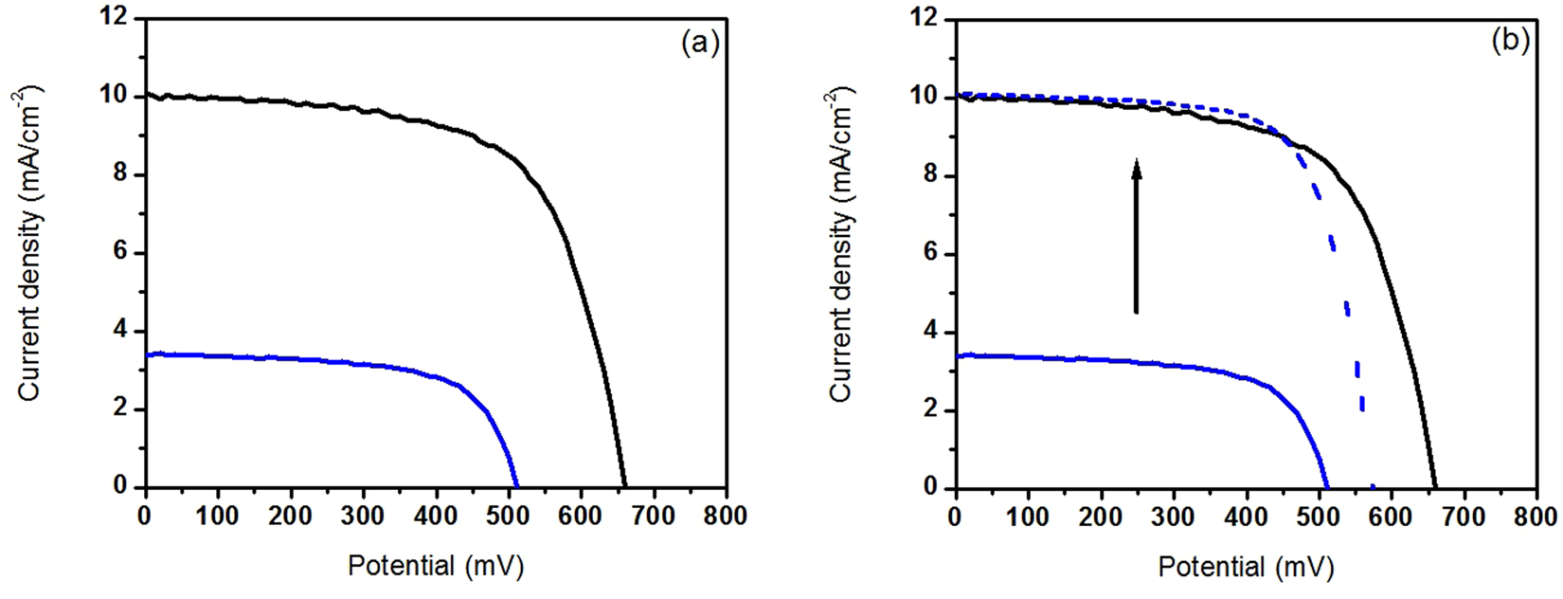
Continuous lines represent J–V curves of **BI54** (blue) and **N719** (black), while the dashed line plots the J–V curve of **BI54** shifted upwards until J_*SC*_ matches that of **N719**.

It is also worth noting that recombination resistances R_REC_ (Fig. 6b and c) of devices sensitized with **N719** and **BI54** show slightly higher value for **N719** (once the as retrieved data, Fig. 6b, are recalculated for the same electron density, Fig. 6c). This finding is not related with the low performance of the devices, as clearly reported by Barea *et al* ^34^. These data suggest that low performances recorded for devices sensitized with **BI54** are ascribable to impaired charge injection from **BI54** LUMO to TiO_2_ CB and are not due to enhanced recombination processes. Indeed, the driving force for charge injection from the LUMO of **BI54** to the CB of the TiO_2_ is reduced compared to the case of **N719** (Fig. 2). By shifting the photocurrent density of **BI54** sensitized cell to simulate the same J_*SC*_ value (namely, similar charge injection condition)^34^ recorded for the **N719** sensitized device, it is possible to observe (Fig. 7) that the two solar energy converting devices perform in the very similar way, confirming that charge injection is the limiting factor for **BI54**.

## Conclusions

Despite the optimistic evaluations pointed out by computational predictions, the push-pull A3B ZnPc **BI54** showed rather low solar to electric power conversion efficiencies once integrated as light harvester in DSSCs. As predicted by models, **BI54** fulfilled the mandatory requirements for energy alignment with respect to TiO_2_ used as ETM and the iodide/iodine redox couple, but the successful implementation of this ZnPc requires a careful control of adsorption conditions and possibly the search for other semiconductors offering a better driving force for charge injection. A major issue with this dye design is its tendency to aggregation, which proved drastically detrimental for cell efficiency. This drawback can be partially alleviated by modulating the sensitization time and using an anti-aggregation agent such as DCA that acts as a molecular spacer on the ETM surface. Also, theoretical calculations did not take into account the possible shift in the CB of TiO_2_, which was highlighted by our experiments and results in a decrease in charge injection efficiency.

Based on these warnings, the design of new push-pull ZnPcs bearing diarylamino substituents is now underway in our laboratories.

## Methods

### Synthesis of BI54

Commercially available reagents were used as received. 4-(diphenylamino)phthalonitrile **1** and pentyl 3,4-dicyanobenzoate **2** were prepared as described in our preceding works^18,35^. Solvents were purified by standard methods and dried if necessary. Reactions were monitored by thin layer chromatography (TLC) that was conducted on plates precoated with silica gel Si 60-F254 (Merck, Germany). Column chromatography was conducted using silica gel Si 60, 0.063–0.200 mm (Merck, Darmstadt, Germany). UV-Vis measurements in solution were performed on a Nicolet Evolution 500 spectrophotometer (Thermo Electron Corporation). FTIR were performed on a Cary 530 instrument (Agilent Technologies). ESI mass spectra were obtained with a ICR-FTMS APEX II (Bruker Daltonics) mass spectrometer. MALDI mass spectra were obtained with a TOF-TOF mass spectrometer Microflex LT (Bruker). Elemental analyses were carried out by the Departmental Service of Microanalysis (University of Milano).

### ZnPc BI54

In a flame dried Schlenk tube equipped with a stir bar a mixture of phthalonitriles **1** (266 mg, 0.90 mmol), **2** (73 mg, 0.30 mmol) and Zn(OAc)_2_.2H_2_O (55 mg, 0.16 mmol) was suspended in dimethylaminoethanol (12 mL). The mixture was stirred for 24 h at 150 °C under a nitrogen atmosphere. The solvent was then evaporated under vacuum and the residue was treated with MeOH (50 mL). The precipitated black solid was collected by filtration on a Büchner funnel and thoroughly washed with MeOH. The raw material was purified twice by column chromatography (silica gel, THF) to give the dimethylaminoethyl ester of **BI54** (MW = 1194) as determined by MALDI-TOF mass analysis (32 mg, 17% with respect to the limiting Zn(II) reagent). The ester was dissolved in THF (4 mL) and treated with 1 M aqueous KOH (1 mL). The mixture was stirred at 70 °C for 8 h, then overnight at room temperature. The organic solvent was removed under reduced pressure and H_2_O (1 mL) was added to the residue. The pH of the aqueous phase was adjusted to 3 by dropwise addition of aqueous 10% HCl and the mixture was extracted twice with DCM (5 mL). The combined organic layers were washed with H_2_O, brine and dried over MgSO_4_. Evaporation of the solvent under reduced pressure afforded the title compound as a mixture of positional isomers (22 mg, 71%). UV-Vis (THF): λ_max_/nm (log ε) = 359 (5.01), 496 (4.37), 640 (4.71), 711 (5.35). FTIR (KBr): υ_max_/cm^−1^ = 3429, 2922, 1679, 1613, 1590, 1490, 1446, 1385, 1331, 1260, 1088, 1046, 745, 695. MS (MALDI-TOF): m/z calcd for (C_69_H_43_N_11_O_2_Zn)^+^ 1121.3. Found: 1121.7. Anal. calcd for C_69_H_43_N_11_O_2_Zn: C 73.76, H 3.86, N 13.71. Found C 73.70, H 3.89, N 13.67.

### Dye-sensitized solar cells

TiO_2_ paste (20 nm anatase nanoparticles 18NR-T) was purchased from Dyesol and multi-walled carbon nanotubes (average length 5–20 μm) were purchased from Sigma-Aldrich. The redox electrolyte was composed of 0.1 M LiI, 0.05 M I_2_, 0.6 M 1,2-dimethyl-3-*n*-propylimidazolium iodide, and 0.5 M 4-*tert*-butylpyridine dissolved in acetonitrile. TiO_2_ paste with MWCNT was prepared by mixing an appropriate amount of TiO_2_ paste (0.102 g) 0.002 wt% of ethanolic solution of MWCNT (0.004 g) under vigorous stirring. Detailed preparation of the paste and deposition of electrodes is reported in ref.^19^.

### Device fabrication

TiO_2_ paste was deposited onto FTO glass (sheet resistance 10 Ω/□) by tape casting technique. The paste was dried 15 min at ambient conditions and then fired on hot plate for 6 min (130 °C). Two layers of transparent TiO_2_ and one of scattering TiO_2_ were deposited. All the samples were finally annealed at 500 °C for 30 min under ambient atmosphere. Photoanode thickness for all the photoanodes was about 15 μm and was evaluated by stylus profilometry.

**BI54** dye was dissolved in tetrahydrofuran and the sensitization of photoanodes was performed for 20, 13, 5 and 4 hours in the dark. Concentration of **BI54** was kept constant in all the experiments at 5.0 × 10^−4^ M; DCA was used at the same concentration.

DSSCs were fabricated using dye sensitized oxide photoanodes and platinized FTO glass as a counter electrode (5 nm Pt thin film deposited by sputtering) separated by 25 μm-thick plastic spacers (Surlin from Solaronix). Electrolyte composition was as it follows: 0.1 M LiI, 0.05 M I_2_, 0.6 M 1,2-dimethyl-3-n-propylimidazolium iodide, and 0.5 M 4-tert-butylpyridine dissolved in acetonitrile. All chemicals were purchased from Sigma–Aldrich and used as received.

### Characterization

UV-Visible measurements were carried out in a T80 spectrophotometer (PG Instruments); quartz cuvettes were used for liquid samples (1 cm optical path). UV-Visible diffuse reflection (DR) spectra on the powders were measured with a Thermo Fisher Evolution 300 spectrophotometer equipped with a DRA-EV-300 integrating sphere. The current–voltage (I-V) characteristics of the fabricated cells were measured without masking by using a Keithley 2400 SourceMeter under simulated sunlight using an ABET2000 solar simulator at AM 1.5 G (100 mW cm^−2^) calibrated using a reference silicon cell. The electrochemical impedance spectroscopy (EIS) was carried out in dark conditions using a SOLARTRON 1260 A Impedance/Gain-Phase Analyzer, with an AC signal 20 mV in amplitude, in the frequency range between 100 mHz and 300 kHz. External bias applied was from 0 to 100 mV above the V_*OC*_.

Electron lifetime of the photogenerated charges present in the CB of the dyed semiconductor is calculated from open circuit voltage decay using the following equation:^36^

3 $${\tau }_{n}=-\frac{{k}_{B}T}{e}{(\frac{d{V}_{OC}}{dt})}^{-1}\,$$

where k_B_T is the thermal energy, e is the positive elementary charge.

## Electronic supplementary material

Supplementary Information
