# Supplementary material for: Dye-sensitized solar cells based on a push-pull zinc phthalocyanine bearing diphenylamine donor groups: computational predictions face experimental reality
Source: Sci Rep. 2017 Nov 15;7:15675. doi: 10.1038/s41598-017-15745-3 (PMC5688058; doi:10.1038/s41598-017-15745-3)
Supplement: Supplementary file 1 — Supplementary Information [file 41598_2017_15745_MOESM1_ESM.doc]

**D****ye-sensitized solar cells based on a push-pull zinc phthalocyanine bearing diphenylamine donor groups: computational predictions face experimental reality**

**Riccardo Milan^a,b,c^, Gurpreet Singh Selopal^a,d,e^ Marco Cavazzini^f^, Simonetta Orlandi^f^, Rita Boaretto^g^ , Stefano Caramori^g^, Isabella Concina*^a,b,h^, Gianluca Pozzi*^f^**

^a^ Department of Information Engineering, University of Brescia, Via Valotti, 9 – 25131 Brescia, Italy

^b^ CNR-INO SENSOR Laboratory, via Branze 45 – 25123 Brescia, Italy

^c^ Schulich Faculty of Chemistry, Technion−Israel Institute of Technology, 32000 Haifa, Israel

^d^ Institute of Fundamental and Frontier Sciences, University of Electronic Science and Technology of China, Chengdu 610054, P. R. China.

^e^ Institut National de la Recherche Scientifique, Centre Énergie, Matériaux et Télécommunications, 1650 Boul. Lionel Boulet, Varennes QC J3X 1S2, Canada.

^f^ Institute of Molecular Science and Technology, ISTM-CNR, Via Golgi 19, 20133 Milano, Italy

^g^ Department of Chemical and Pharmaceutical Sciences, University of Ferrara, Via Borsari 46, 44121 Ferrara, Italy

^h^ Division of Materials Science, Department of Engineering Sciences and Mathematics, Luleå University of Technology, 971 87 Luleå, Sweden

* Corresponding author. e-mail addresss: [isabella.concina@ltu.se](mailto:isabella.concina@ltu.se)

* Corresponding author. e-mail addresss: [gianluca.pozzi@istm.cnr.it](mailto:gianluca.pozzi@istm.cnr.it)

**Supporting Information**

**Scheme S1. Synthesis of BI54.**

**Figure S1. UV-Vis absorption spectra of BI54 in DCM (black line) and THF (red line).**

**Figure S2. Normalized absorption (black line) and emission (blue line) spectra of BI54 in THF.**

**Electrochemical characterization**

Electrochemical characterizations were performed through an Autolab PGSTAT30 (Ecochemie) potentiostat/galvanostat interfaced with a PC under GPES software. For cyclic voltammetry, a three electrode, one compartment electrochemical cell was used. The reference electrode was SCE (with saturated KCl solution); the auxiliary electrode was Pt and the working electrode was glassy carbon. The working electrode was polished subsequently with 1 and 0.3 mm alumina powder and then rinsed with distilled water. All experiments were carried out in THF (Aldrich) using 0.1 M tetrabutylammonium hexafluorophosphate (TBAPF_6_, Fluka, for electrochemical analysis 99%). The electrolyte solution was degassed with nitrogen prior to measurements. All redox potentials were referenced against SCE. Peak potentials were measured at scan rates variable from 0.02 Vs^-1^ to 3 Vs^-1^.

|  |  |
| --- | --- |
|  |  |

Figure S3. Electrochemical analyses of dye BI54. (a) Cyclic voltammetry recorded in THF/0.1 M TBAPF_6_ with anodic (black) and cathodic (red) regions separately scanned; scan rate 0.1 V/s.( WE:glassy carbon, CE:Pt, RE:SCE). (b) Cyclic voltammetry recorded in THF at different scan rates (from 0.02 V/s to 3V/s) (WE:glassy carbon, CE:Pt, RE: SCE). (c) anodic peak current vs
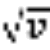
. (d) Anodic vs Cathodic peak current ratio as a function of
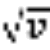
.

The heterogeneous electron transfer kinetics at glassy carbon interfaces resulted in quasi-reversible, diffusion-limited waves, with chemical reversibility attained for scan speed > 100 mV/s.

| Dye | λ_abs_(nm)  (ε)(10^4^ M^-1^cm^-1^) | λem (nm) | E^00^(eV)^[a]^ | E_(HOMO)_ vs NHE(V) (vs vacuum) (eV)^[b]^ | E_(LUMO)_ vs NHE (V) (vs vacuum) (eV) ^[c]^ |
| --- | --- | --- | --- | --- | --- |
| BI54 | 495  641  710 | 765 | 1.68 | 0.905 (-5.4) | -0.77 (-3.72) |

Table S1. Optical and electrochemical properties of the BI54 dye recorded in THF. [a] Calculated from the intersection of normalized absorption and emission spectra. [b] Evaluated from oxidation potential obtained from CV, using -4.5 eV potential for NHE vs. vacuum. [c] Obtained from E^00^ – E_HOMO_.
